# Altered levels of dopamine transporter in the frontal pole and dorsal striatum in schizophrenia

**DOI:** 10.1038/s41537-019-0087-7

**Published:** 2019-12-02

**Authors:** Hirotaka Sekiguchi, Geoff Pavey, Brian Dean

**Affiliations:** 1 Okehazama Hospital Fujita Mental Care Centre, Toyoake, Japan; 2 Department of Psychiatry, Nagoya University Graduate School of Medicine, Nagoya, Japan; 3 The Florey Institute of Neuroscience and Mental Health, Parkville, VIC Australia; 4 The Centre for Mental Health, the Faculty of Health, Arts and Design, Swinburne University, Hawthorne, VIC Australia

**Keywords:** Molecular neuroscience, Schizophrenia

## Abstract

The dopamine hypothesis proposes that there is a hypodopaminergic state in the prefrontal cortex and a hyperdopaminergic state in the striatum of patients with schizophrenia. Evidence suggests the hyperdopaminergic state in the striatum is due to synaptic dopamine elevation, particularly in the dorsal striatum. However, the molecular mechanisms causing disrupted dopaminergic function in schizophrenia remains unclear. We postulated that the dopamine transporter (DAT), which regulates intra-synaptic dopamine concentrations by transporting dopamine from the synaptic cleft into the pre-synaptic neuron, could be involved in dopaminergic dysfunction in schizophrenia. Therefore, we measured levels of DAT in the cortex and striatum from patients with schizophrenia and controls using postmortem human brain tissue. Levels of desmethylimipramine-insensitive mazindol-sensitive [^3^H]mazindol binding to DAT were measured using in situ radioligand binding and autoradiography in gray matter from Brodmann’s area (BA) 10, BA 17, the dorsal striatum, and nucleus accumbens from 15 patients with schizophrenia and 15 controls. Levels of desmethylimipramine-insensitive mazindol-sensitive [^3^H]mazindol binding were significantly higher in BA 10 from patients with schizophrenia (*p* = 0.004) and significantly lower in the dorsal striatum (dorsal putamen *p* = 0.005; dorsal caudate *p* = 0.007) from those with the disorder. There were no differences in levels of desmethylimipramine-insensitive [^3^H]mazindol binding in BA 17 or nucleus accumbens. These data raise the possibility that high levels of DAT in BA 10 could be contributing to lower synaptic cortical dopamine, whereas lower levels of DAT could be contributing to a hyperdopaminergic state in the dorsal striatum.

## Introduction

Schizophrenia has a wide range of symptoms that includes hallucinations, delusions, disorganized thinking, anhedonia, and apathy.^1^ These core symptoms are classified into groupings that are known as positive, negative, and cognitive symptoms. Significantly, the molecular basis of schizophrenia has yet to be fully elucidated but one long-standing proposal is the dopamine hypothesis, which posits there is a hyperdopaminergic state in the mesolimbic system that is critical in generating the positive symptoms of schizophrenia.^2^ In addition, there is a hypodopaminergic state in the mesocortical pathway, which is thought to contribute to negative and cognitive symptoms.

A role for a hyperdopaminergic state in the mesolimbic system is supported by data from neuroimaging studies. Thus, studies using single photon emission computerized tomography, which took the reduction [^123^I](S)-(-)-3-iodo-2-hydroxy-6-methoxy-N-[(1-ethyl-2-pyrrolidinyl) methyl]benzamide ([^123^I]IBZM) binding to the dopamine D2 receptor as an indirect measure of dopamine release following the administration of amphetamine, reported increased levels of dopamine release in patients with schizophrenia.^3,4^ This hypothesis has been supported by a meta-analysis^5^ and the increase in dopamine release in schizophrenia is of a relatively high effect size.^6^ Another study used positron emission tomography (PET) and 3,4-dihydroxy-6-[(^18^)F]fluoro-l-phenylalanine ((^18^)F-DOPA) to measure dopamine synthesis capacity to show that there is an elevated dopamine synthesis capacity in the striatum, and in particular the associative but not the limbic striatum, in those at ultra-high risk for psychosis.^7^ These data suggested the associate striatum as a key region for dopaminergic dysregulation very early in the onset of the disorder. In a follow-up study it was shown that individuals that developed a psychotic disorder had the greatest dopamine synthesis capacity in the associative striatum and that there was a positive correlation between dopamine synthesis capacity and symptom severity in those that developed a psychotic disorder.^8^ These data supported the notion of a hyperdopaminergic state in the striatum as being important in the genesis of psychotic symptoms in schizophrenia. Another PET neuroimaging study used carbon 11-labeled FLB 457 binding to dopamine D2-like receptors^9^ after an amphetamine challenge to show a reduced release of dopamine in the dorsolateral prefrontal cortex in patients with schizophrenia.^10^ These data support the proposition of a hypodopaminergic state in the cortex in patients with the disorder.

Whilst neuroimaging studies strongly support the presence of changed dopaminergic states in the CNS from subjects with schizophrenia, they do not aid in the identification of the molecular mechanisms that cause changes in dopaminergic activity in either the striatum or cortex. In considering potential molecular mechanisms involved in abnormal dopamine neurotransmission in schizophrenia, it is significant that the dopamine transporter (DAT: gene name = Solute Carrier Family 6 Member 3; SLC6A3) is located on the presynaptic membrane of dopaminergic terminals and regulates dopamine neurotransmission by removing dopamine from the synaptic cleft.^11^ Studies using DAT knock out mice have shown these animals display a persistent increase in extracellular dopamine,^12,13^ which adds to the argument that DAT is an important regulator of synaptic dopamine concentration. Notably, two meta-analyses of neuroimaging measuring DAT concluded there were no significant changes in levels of DAT in the striatum of patients with schizophrenia.^5,14^ These findings agree with some postmortem brain studies using tissue homogenates that have reported no changes in DAT in the striatum from patients with schizophrenia.^15,16^. By contrast, using in situ radioligand binding and autoradiography, our laboratory has repeatedly shown that there were significantly lower levels of DAT in the striatum^17,18^ but that there was no change in DAT in Brodmann’s area (BA) 9.^19^

There are some important considerations that have impacted on the potential mechanisms by which DAT could be involved in the pathophysiology of schizophrenia. Firstly, the striatum has been divided into the dorsal (caudate and putamen) and ventral striatum (nucleus accumbens).^20^ Functionally, the dorsal striatum is connected to the motor cortex and the dorsolateral frontal cortex, whereas the ventral striatum is connected to the medial and orbital frontal cortex. In addition, the dorsal striatum can be clearly divided into the dorsal putamen and the dorsal caudate which are included in what is termed the associative striatum.^21^ Importantly, neuroimaging studies have shown that elevated dopamine synthesis in schizophrenia is most apparent in the associative striatum.^2^ Additionally, we have recently shown that changes in gene expression are more abundant in the frontal pole than they are in the dorsolateral prefrontal cortex from patients with schizophrenia.^22^ This finding is significant as the frontal pole is highly developed in humans,^23^ is involved in higher cognitive functions^24^ and impaired cognitive function has been related to atrophy of the frontal pole.^25,26^

Given the important role of DAT in maintaining dopamine homeostasis, the anatomical complexity of the striatum and our recent data showing abundant changes in gene expression in the frontal pole from patients with schizophrenia,^22^ we decided to measure levels of DAT, using desmethylimipramine-insensitive mazindol-sensitive [^3^H]mazindol binding and autoradiography, in the frontal pole (BA 10) and the striatum from patients with schizophrenia. We also measured levels of DAT in the visual cortex (BA 17) as a negative control as this is a cortical region involved in the pathophysiology of schizophrenia^27,28^ but there is no strong evidence for disrupted cortical neurotransmission in that cortical region.^29^ We chose to use [^3^H]mazindol, rather than another radioligand that would bind to DAT, so we could more readily relate our findings to our previous studies on DAT in schizophrenia in which we used that radioligand.^17,18^ The outcome of our cortical studies would be of added interest because our expression microarray experiments lacked the sensitivity to detected DAT mRNA.

## Results

### Demographic, CNS collection and CNS related

There were no statistically significant differences in age, gender frequency, PMI or CNS pH with diagnoses (Table 1).

**Table 1:** A summary of the demographic, pharmacological and CNS related data (mean ± SEM) for the 15 donors with schizophrenia and 15 controls used in this study

	Age (yr)	Gender (M/F)	PMI (h)	pH	DI	FRADD	LEAP
Controls	60 ± 3.9	7/8	44 ± 4.2	6.30 ± 0.07			
Schizophrenia	59 ± 3.0	7/8	44 ± 2.9	6.33 ± 0.05	29 ± 2.9	379 ± 74	1110 ± 272
*p*	0.92	1.00	0.87	0.64			

*DI* duration of illness, *F* female, *FRADD* final recorded antipsychotic drug dose expressed as chlorpromazine equivalents per day, *LEAP* lifetime exposure to antipsychotic drugs expressed as chlorpromazine equivalents/10,000, *PMI* postmortem interval, *M* male, *yr* year

### Human CNS

**Fig. 1:**
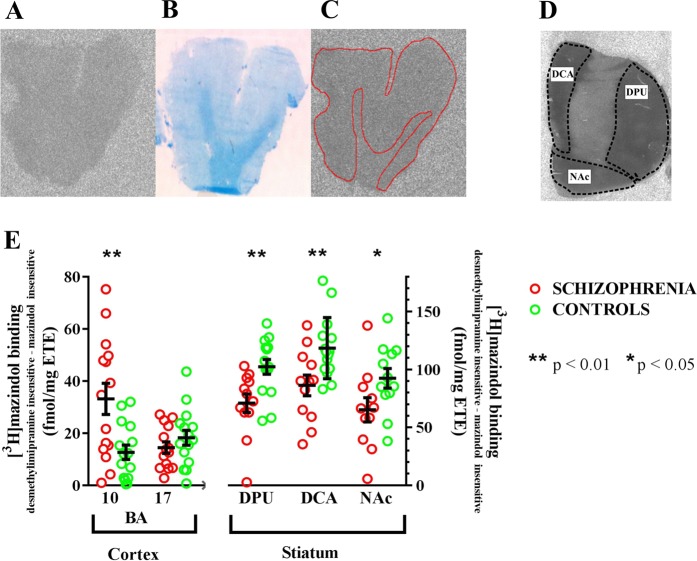
**a** A typical autoradiograph showing desmethylimipramine-insensitive mazindol-sensitive [^3^H]mazindol binding to Brodmann’s area (BA 10). **b** The section used to generate the autoradiograph shown A after Klüver–Barrera staining to show white matter, **c**: The autoradiograph in **a** with the gray matter outlined in reference to what was observed as white matter in the section in **b**. **d** A typical autoradiograph from the striatum partitioned into the dorsal putamen (DCU), the dorsal caudate (DCA), and the nucleus accumbens (NAc). **e** Desmethylimipramine-insensitive mazindol-sensitive [^3^H]mazindol binding (showing mean ± SEM) to Brodmann’s (BA) area 10 and 17, the DCU, DCA, and NAc from patients with schizophrenia and controls

In the cortex, all desmethylimipramine-insensitive mazindol-sensitive [^3^H]mazindol binding was shown to have a normal distribution (d’Agostino and Pearson omnibus normality test: *K*2 = 1.59 to 2.39; *p* = 0.30–0.45) and, compared to controls, the level of desmethylimipramine-insensitive mazindol-sensitive [^3^H]mazindol binding was higher in BA 10 (Student’s *t*-tests: *t* = 3.114, *p* = 0.004, Cohen’s *d* = 1.109), but not BA 17 (Student’s *t* tests: *t* = 1.042, *p* = 0.307, Cohen’s *d* = 0.412), from the patients with schizophrenia (Fig. 1e). The difference in desmethylimipramine-insensitive mazindol-sensitive [^3^H]mazindol binding in BA 10 was at a level of significance that survived Bonferroni correction for multiple measures (i.e. *p* ≤ 0.01). There were no correlations between the binding of desmethylimipramine-insensitive mazindol-sensitive [^3^H]mazindol in BA 10 with that in BA 17, dorsal caudate or nucleus accumbens but there was a weak correlation between desmethylimipramine-insensitive mazindol-sensitive in BA 10 and the dorsal putamen (Supplementary Table 2). There were no correlations between desmethylimipramine-insensitive mazindol-sensitive [^3^H]mazindol in BA 17 and any region in the striatum.

In the striatum, all desmethylimipramine-insensitive mazindol-sensitive [^3^H]mazindol binding for the controls and patients with schizophrenia were normally distributed (d’Agostino and Pearson omnibus normality test: *K*2 = 0.20–3.50; *p* = 0.08–0.90) except for the data from the dorsal putamen from patients with schizophrenia, which was not normally distributed (d’Agostino and Pearson omnibus normality test: *K*2 = 0.76; *p* = 0.02). Given the overwhelming amount of our data was normally distributed we decided to analyze all data using parametric statistics. Thus, striatal desmethylimipramine-insensitive mazindol-sensitive [^3^H]mazindol binding was lower in the dorsal putamen (Student’s *t* tests: *t* = 3.123, *p* = 0.005, Cohen’s *d* = 1.189), dorsal caudate, (Student’s *t* tests: *t* = 2.013, *p* = 0.007, Cohen’s *d* = 1.119) but not the nucleus accumbens (Student’s *t* tests: *t* = 2.045, *p* = 0.054, Cohen’s *d* = 0.854), from patients with schizophrenia (Fig. 1e); differences in desmethylimipramine-insensitive mazindol-sensitive [^3^H]mazindol binding in the dorsal putamen and dorsal caudate were at a level of significance that survived Bonferroni correction for multiple measures.

The linear correlations between desmethylimipramine-insensitive mazindol-sensitive [^3^H]mazindol binding in the dorsal putamen with the dorsal caudate and nucleus accumbens, as well as between desmethylimipramine-insensitive mazindol-sensitive [^3^H]mazindol binding in the dorsal caudate and nucleus accumbens accommodated a lot of the variance between these pairs of data (Supplementary Table 2).

**Fig. 2:**
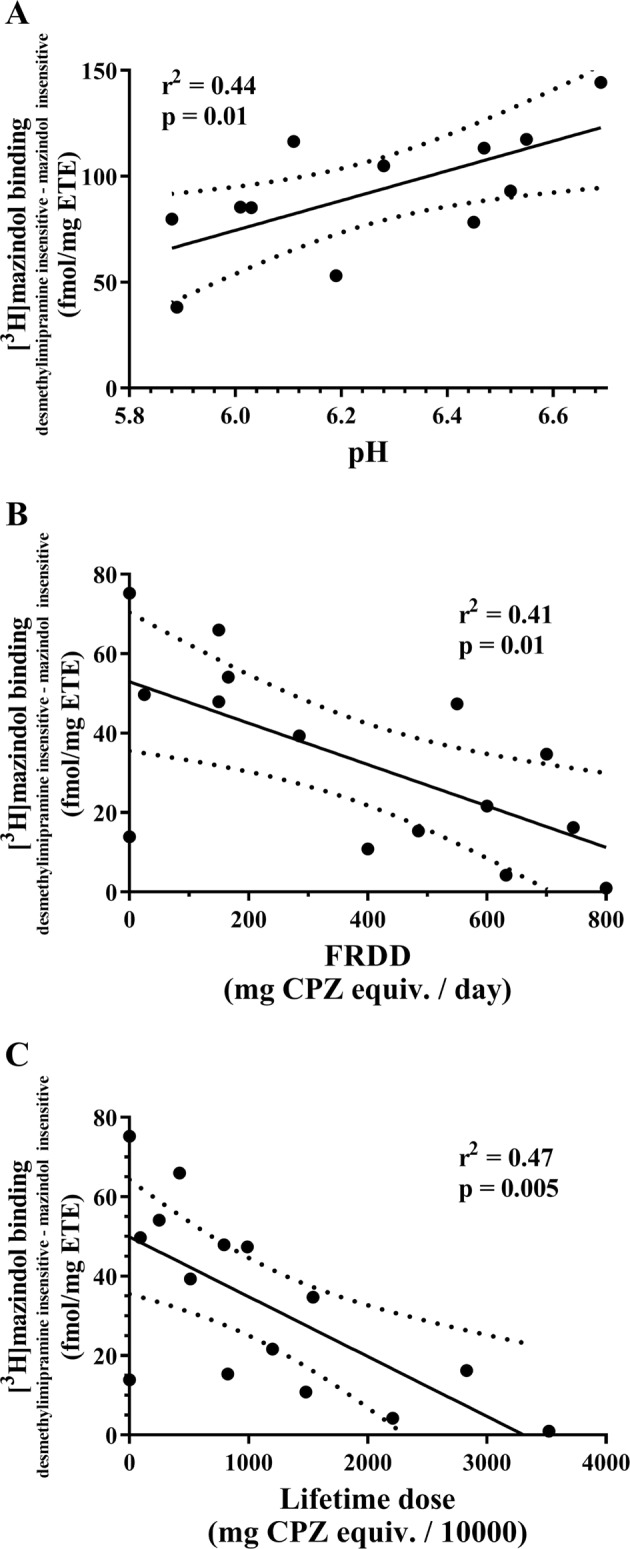
The relationship between desmethylimipramine-insensitive mazindol-sensitive [^3^H]mazindol binding in the: **a** nucleus accumbens from controls and CNS pH, **b** Brodmann’s area 10 from patients with schizophrenia and final recorded antipsychotic drug dose (FRADD) and **c** Brodmann’s area 10 from patients with schizophrenia and lifetime exposure to antipsychotic drugs (LEAP). Solid line = best fit linear regression, dotted line = 95% confidence limits

Exploring data for potential confounds, it was notable that correlations between desmethylimipramine-insensitive mazindol-sensitive [^3^H]mazindol binding and the demographic, CNS collection or pharmacological data did not exceed *r*^2^ = 0.49 (Supplementary Table 3). However, the relationship between desmethylimipramine-insensitive mazindol-sensitive [^3^H]mazindol in nucleus accumbens and pH in controls (Fig. 2a), as well as desmethylimipramine-insensitive mazindol-sensitive [^3^H]mazindol in BA 10 and FRADD (Fig. 2b), as well as LEAP (Fig. 2c) approached *r*^2^ = 0.49. Therefore, secondary analyses of variance were carried out using the potential covariates in ANCOVAs. These analyses showed that CNS pH was not a significant covariate (ANCOVA *p* = 0.44) and that, whilst the levels of desmethylimipramine-insensitive mazindol-sensitive [^3^H]mazindol in nucleus accumbens between patients with schizophrenia and controls did differ (ANCOVA *p* < 0.048), this difference did not survive a Bonferroni correction. By contrast, whilst FRADD (ANCOVA *p* = 0.001) and LEAP (ANCOVA *p* < 0.001) were both significant covariates, the difference between desmethylimipramine-insensitive mazindol-sensitive [^3^H]mazindol in BA 10 from subjects with schizophrenia remained significantly different from that in controls (ANCOVA *p* < 0.0001 for both FRADD and LEAP), a level of significance that survived Bonferroni correction. Notably, these data add to the notion that, in small cohorts where relationship are measured using linear regression, relationships where *r*^2^ < 0.49 does not warrant a secondary analysis.

### Rat CNS

The liquid intake by all groups of rats was consistent with their receiving the expected daily dose of antipsychotic drugs administered in drinking water.^30^

There were no significant differences in desmethylimipramine-insensitive mazindol-sensitive [^3^H]mazindol binding to the frontal cortex of rats treated with varying doses of haloperidol for 3 months (Supplementary Fig. 1a) or in the cortex or striatum from rats treated with varying doses of thioridazine for the same period of time (Supplementary Fig. 1c, d). By contrast, compared to vehicle, the level of desmethylimipramine-insensitive mazindol-sensitive [^3^H]mazindol binding to striatum was higher in rats treated with 0.01 mg/kg/day of haloperidol for 3 months (Supplementary Fig. 1b); this difference from vehicle was not apparent at higher doses of haloperidol (Supplementary Fig. 1b).

## Discussion

In this study we report, compared to controls, higher levels of desmethylimipramine-insensitive mazindol-sensitive [^3^H]mazindol binding in BA 10 and lower levels of desmethylimipramine-insensitive mazindol-sensitive [^3^H]mazindol binding in the dorsal putamen and dorsal caudate in patients with schizophrenia. Importantly, under the conditions used in this study, desmethylimipramine-insensitive mazindol-sensitive [^3^H]mazindol binding would predominantly bind to DAT^31–35^ and therefore our data argues that levels of DAT are higher in BA 10 and lower in areas of the striatum from people with schizophrenia. However, it needs to be acknowledged that the data defining the selectivity of desmethylimipramine-insensitive mazindol-sensitive [^3^H]mazindol binding was generated using rodent CNS or cellular models. Hence it is possible that there may be sites other than DAT that are contributing to the levels of desmethylimipramine-insensitive mazindol-sensitive [^3^H]mazindol binding in human CNS. In addition, levels of noradrenaline transporters in the human cortex are higher than those in the striatum, and therefore it could be possible that not all noradrenaline transported binding is blocked at the concentration of desmethylimipramine used in this study. Against this argument are data showing [^3^H]mazindol binding is totally displaced from the noradrenaline transporter by 0.3 µM desmethylimipramine in the human locus coeruleus, where noradrenaline transporter levels are higher than those in human cortex.^33^

Our finding of increase in DAT in BA 10 but not in BA 17, along with our studies in BA 9^19^ showing no changes in cortical DAT, suggests that changes in cortical DAT in schizophrenia may be limited to BA 10. If changes in DAT levels in BA 10 from patients with schizophrenia are associated with changed dopamine homeostasis, that would be significant as BA 10 is involved in the control of higher cognitive functions^24^ and possibly, the abnormal activation at resting state that has been observed in BA 10 in patients with schizophrenia using fMRI.^36^ Overall, our data argues for a cortical-regionally selective hypodopaminergic state, because of increased dopamine uptake by higher DAT levels in BA 10, that would likely be involved in the cognitive deficits of schizophrenia^37^ because of the important role of BA 10 in maintaining cognitive ability.^38^

Contrasting to our findings in BA 10, our data shows lower levels of desmethylimipramine-insensitive mazindol-sensitive [^3^H]mazindol to DAT in the dorsal putamen and dorsal caudate from patients with schizophrenia. These data are consistent with earlier findings using this approach to measuring DAT in the striatum from patients with the disorder,^17,18,39^ but differ from studies that reported no change in levels of DAT in the caudate nucleus that use other radioligand to measure levels of the dopamine transporter.^15,16^ Notably, two of these studies,^15,16^ reported radioligand binding to membrane homogenates made from all of the caudate nucleus and the study using autoradiography did not attempt to subdivide the striatum into sub-regions. Two studies had much smaller diagnostic cohorts.^16^ Hence there are a number of methdological considerations that could be contributing to the differences in our data and those from studies using other radioligands.

In interpreting our postmortem CNS data, current data suggest that lower levels of DAT would be consistent with the presence of higher levels of extracellular dopamine.^12,13^ Hence, our findings on DAT could be synergistic with findings suggesting elevated dopamine synthesis capacity in patients with schizophrenia and the conclusion that high levels of dopamine in the striatum in patients with schizophrenia are contributing to the onset of positive symptoms.^40^

It has been suggested that hypoactive dopamine neurotransmission in prefrontal cortex leads to disinhibition of subcortical mesolimbic dopamine activity, resulting in hyperstimulation of the dopamine D2 receptor and positive symptoms of schizophrenia.^2^ Conversely, it has been argued that increased dopamine activity at the dopamine D2 receptor in the striatum might reduce the ability of the dorsolateral prefrontal cortex to engage striatal processing functions, resulting in blunted cortical dopamine release based on the study of rats whose dopamine D2 receptor was selectively over-expressed.^41^ Notably, we found there was no correlations between levels of DAT in BA 10 and striatum, hence our data does not suggest linked changes in DAT across regions or informs as to whether changes in cortical or sub-cortical DAT represents a primary lesion.

There are limitations to our study. As in any study of schizophrenia using patients who have been medicated, the effects of antipsychotic drugs on DAT could be a confound. We found significant negative correlation between final recorded antipsychotic drug dose (FRADD), as well as lifetime exposure to antipsychotic drugs (LEAP) and DAT in BA 10. Thus, it is possible that differences in levels of DAT between patients with schizophrenia and controls could be greater in BA 10 from medication naïve individuals, a notion supported by an increase in the significance in the difference between desmethylimipramine-insensitive mazindol-sensitive [^3^H]mazindol binding in BA 10 from subjects with schizophrenia if FRADD or LEAP was included as a covariate.

In this study, we also reported higher levels of desmethylimipramine-insensitive mazindol-sensitive [^3^H]mazindol binding in the striatum of rats treated with 0.01 mg/kg/day, but not higher doses, of haloperidol. Our findings have some similarities with an earlier study that found no change in the levels of [^3^H]GBR 12,935 to the dopamine transporter in the striatum of rats treated with haloperidol at 1 mg/kg/day for 21 days,^42^ and a study that showed no changes in [^125^I]RTI-121 binding to the striatal dopamine transporter after treating rats with either 1 mg/kg haloperidol or 20 mg/kg clozapine per day.^43^ These data are consistent with the hypothesis that treating rats with “clinically equivalent” doses of haloperidol^44^ does not affect level of radioligand binding to the dopamine transporter in the striatum. Notably, in our study, the dose of haloperidol that lowered levels of DAT in rat striatum would be viewed as being equivalent to a sub-clinical dose in humans^45,46^ and therefore may not be relevant to doses received by the patients with schizophrenia in this study. This posit is supported by an imaging study which reported no change in DAT in patients with schizophrenia after 6 months of treatment with haloperidol,^47^ and another study that showed existing lower striatal DAT levels in patients with schizophrenia did not change after 4-week treatment with antipsychotic drugs.^48^ Thus, overall current data from rat studies suggests that antipsychotic drug treatment is not likely to be a major confound in our study. However, an in vivo electrophysiology study has reported that acute treatment with 10 µM haloperidol, but not clozapine, reduced striatal dopamine uptake in the striatum.^49^ In addition, studies using synaptosomes from rodent striatum have reported that metabolites of haloperidol, but not haloperidol itself, can inhibit dopamine uptake.^50,51^ Thus, whilst treatment with drugs such as haloperidol may not alter levels of the dopamine transporter, it could alter levels of dopamine uptake by the transporter.

In humans, neuroimaging studies have reported lower^52^ or no change in radioligand binding to the dopamine transporter in drug naïve patients with schizophrenia.^14,47,^^53–55^ By contrast, one of these groups,^56^ and another study,^57^ found there were lower levels of radioligand binding to the striatal dopamine transporter in treated patients with schizophrenia. These data could be interpreted as showing lower levels of striatal dopamine transporters in schizophrenia have resulted from antipsychotic drug treatment. However, one neuroimaging study^56^ and one postmortem study^39^ have reported a differential loss of radioligand binding to the dopamine transporter with age in subjects with schizophrenia. This has led to the argument that a loss of striatal dopamine transporter is associated with disease progression in patients with schizophrenia rather than an outcome from antipsychotic drug treatment.

While our case history reviews, or extensive clinical details in histories, are often limited, meaning it is not possible to relate our findings to, for example, symptom severity. In addition, because typical drugs were chosen for our studies in animals, we have no data on the impact of atypical antipsychotic drugs on levels of DAT.

In conclusion, our data raise the possibility that high levels of DAT in BA 10 could be causing low synaptic cortical dopamine concentration, whereas lower levels of dorsal striatal DAT could contribute to a hyperdopaminergic state in that CNS region. Therefore, this might be the first study to show at least part of the molecular mechanisms that could lead to a hypodopaminergic state in the cortex and hyperdopaminergic state in the striatum of patients with schizophrenia.

## Methods

### Ethical approval

Approval to collect human tissue was obtained from the Ethics Committee of the Victorian Institute of Forensic Medicine with written permission to collect tissue being obtained from the next of kin empowered to give such permission under law in the State of Victoria. Approval for the use of animals for these studies was from the Animal Ethics Committee of the Florey Neurosciences Institute.

### Tissue collection

After CNS collection at autopsy, the left hemisphere from each donor was processed in a standardized manner allowing tissue to be frozen to −80 °C within 30 min of autopsy.^19^ CNS pH^58^ was measured for each individual as an indicator of the quality of tissue preservation.^59^

### Case history review

For each psychiatric case, relevant data from clinical histories and interviews with treating clinicians and relatives was obtained using a standardized instrument, the Diagnostic Instrument for Brain Studies.^60^ The information collected allowed the diagnosis of schizophrenia to be made by consensus using DSM-IV criteria.^61^ Controls were individuals where no history of psychiatric illness could be found from medical records, their treating clinician, or family members. Postmortem interval (PMI) was calculated as either the time from witnessed death to autopsy or the time mid-way between a subject being last seen and being found dead until autopsy; tissue was only collected from individuals who had been seen within 5 h before being found dead. Duration of illness (DI) was calculated as the time from first presentation to a medical facility, where a psychiatric diagnosis was recorded to death. The FRADD was recorded and standardized to doses expressed as mg chlorpromazine equivalents per day.^62^ The recorded doses of antipsychotic drugs across the whole DI were also converted to mg chlorpromazine equivalents and summed to give a LEAP, which were expressed as chlorpromazine equivalents/10,000.

### Human brain processing

All human tissues were sourced through the Victorian Brain Bank Network, the Florey Institute for Neuroscience and Mental Health, Australia. Tissue, from the left hemisphere, was collected from BA 10 (most rostral portions of the superior frontal gyrus and middle frontal gyrus, bounded ventrally by the superior rostral sulcus), BA 17 (the region in the occipital cortex in which the band of Gennari is evident) and striatum from 15 patients with schizophrenia and 15 individuals with no history of psychiatric or neurological illness (controls) (Table 1; Supplementary Table 1). None of the subjects in this study had been included in our previous studies of [^3^H]mazindol binding in schizophrenia.^17,18^

### Antipsychotic drug treatment in rats

Using a well-described protocol,^30^ groups of 6-week-old Sprague-Dawley rats (*n* = 5–7 per group) were treated with vehicle (0.9% isotonic saline solution), haloperidol (0.01, 0.1 mg/kg, or 1.0 mg/kg/day) or thioridazine (0.1, 1.0 mg/kg, or 10 mg/kg/day) for 3 months in drinking water. From the recorded consumption of water per cage and the number of rats per cage, it was calculated that the dose of drug received by each rat would have been within 10% of the target daily intake. These two antipsychotics were used because they have different affinity to dopamine D2 receptor,^63^ and the majority of patients from whom tissue was collected for this study had been treated with first generation antipsychotic drugs or the second generation antipsychotic drug, risperidone, both of which have a limited selective pharmacology, which includes a high affinity for the dopamine D2-like receptors.^64^ Rats were euthanized by sodium pentobarbitone overdose (1.5 ml i.p. injection of 60 mg/ml solution) and brains were rapidly collected, frozen, and stored at −80 °C until required.

### In situ radioligand binding and autoradiography

Using a cryostat, 20 µm frozen sections were cut from human BA 10, BA 17, and striatum. In addition, rodent CNS was sectioned to obtain frozen sections with frontal cortex and striatum (between Bregma −1.30 and 0.2 mm^65^). All sections were thaw mounted on to gelatinized slides. Prior to being incubated with the radioligand, tissue sections were incubated in 50 mM Tris buffer containing 300 mM NaCl, 5 mM KCl for 60 min at 4 °C.^66^

Total [^3^H]mazindol (15 nM; PerkinElmer) binding to DAT (TB: three sections) was taken as the binding of radioligand in the presence of 0.3 µM desmethylimipramine (DMI), a drug concentration which completely displaces [^3^H]mazindol binding from the noradrenaline^31^ and the serotonin^32^ transporter. The difference between the binding of [^3^H]mazindol in the presence of DMI and in the absence and presence of mazindol (10^–6^ M) (NSB: two sections humans; three sections rats) was taken as specific binding to DAT.^33^ After exposure to radioligand, all sections were washed twice in ice-cold assay buffer, dipped into ice-cold distilled water, and then dried thoroughly. The sections where then partially post-fixed overnight in paraformaldehyde fumes before being apposed to a BAS-TR2025 plate (Fujifilm, Japan) with [^3^H]microscales^TM^ (Amersham Biosciences, UK) for 7 days. All plates were scanned in a BAS5000 high-resolution phosphoimager (Fujifilm, Japan). The resulting images were analyzed using AIS imaging software (Imaging Research, Canada).

To measure the intensity of radioligand binding in the cortex, we took an integrated measure of the intensity of radioligand binding across Lamina I–VI, as binding was homogenous across those laminae. However, because the border between gray and white matter was not clearly visible due to the low intensity of DAT in prefrontal cortex (Fig. 1a), the cytoarchitectural boundary between white and gray matter was defined by comparison to the original section stained by Klüver–Barrera staining (Fig. 1b) and transposed onto each autoradiograph (Fig. 1c). In the striatum, each autoradiograph was divided into dorsal caudate and dorsal putamen (Fig. 1d), as there were clear areas of low radioligand binding separating these areas. Results were obtained as disintegrations per minute (DPM) of estimated wet weight tissue equivalents (ETE) and converted to fmol/mg estimate tissue equivalents (ETE).

### Klüver–Barrera staining

For Klüver–Barrera staining, sections were fixed in 10% formalin in phosphate-buffered saline for 1 h at room temperature. Sections were immersed in 95% ethanol to hydrate and subsequently in 0.1% Luxol Fast Blue overnight at 37 °C and then rinsed with 95% ethanol. After rinsing in distilled water, the slides were alternately immersed in 0.05% lithium carbonate for 5–20 s, and in 70% ethanol for 1–2 min to differentiate the stain until the borderline was clear and white matter was sharply defined.

Sections were counterstained with 0.1% Cresyl Violet for 20 min at 37 °C and rinsed in 70% ethanol and 95% ethanol, then dehydrated through two changes of xylene each for 2 min. Sections were mounted in DPX and imaged using a light microscope with a digital camera attached.

### Statistical analyses

First, all groups of data were interrogated with the d’Agostino and Pearson omnibus normality test as this is best at determining data distribution in small cohorts.^67^ For normally distributed experimental data, outliers were identified using the Grubb’s test which allows the exclusion of a single outlier per data set.^68^

For normally distributed data, Student’s *t*-tests were performed to identify variation in data between measures, with *p* < 0.05 being taken as indicating a significant variation. Gender frequency was compared using the Fisher’s exact test. Cohen’s *d* was calculate at https://www.socscistatistics.com/effectsize/default3.aspx.

The low number of animals in our rat study meant it was not possible to accurately assess the distribution of the data. Therefore, an ANOVA was used to identify significant variance and a post hoc Bonferroni comparing all drug doses within CNS region and correcting for multiple comparisons was used to identify the source of any variance; *p* < 0.05 was considered significant.

Relationships between experimental data and between experimental data and demographic, pharmacological and CNS collection data were identified using linear regressions. A linear regression was accepted as deviating from the horizontal when *p* < 0.05. Such an outcome was taken as showing some covariance between two factors, which could require a secondary analysis of variance designed to assess the impact of covariance. Next, the percentage of the variance described by the linear regression line, as shown by the Pearson’s product–moment, was assessed. Notably, there are now significant data showing that in studies such as this that have relatively small cohort size relationships between variables, where *r*^2^ that does not exceed 0.49 does not impact on the outcome of a primary analysis of variance.^69,70^ Therefore, in this study only relationships between variables were *r*^2^ > 0.49 that would be included in an ANCOVA to determine if relationships were impacting on the primary analyses of variance.

When data were not normally distributed, equivalent non-parametric tests would be used with no attempt being made to identify outliers.

All analyses were conducted using GraphPad Prism, except for ANCOVAs, which were conducted using Minitab 18.

### Reporting summary

Further information on research design is available in the Nature Research Reporting Summary linked to this article.

## Supplementary information

Reporting Summary

Supplementary Infomation

## Data Availability

All data is held by the Florey Institute for Neuroscience and Mental Health, reasonable requests to access raw data will be considered if it is judged providing such data would not compromise the privacy of tissue donors.No databases were generated or analyzed during the current study.

## References

[1] Tandon R, Nasrallah HA, Keshavan MS (2009). Schizophrenia, “just the facts” 4. Clinical features and conceptualization. Schizophr. Res..

[2] Howes OD, Kapur S (2009). The dopamine hypothesis of schizophrenia: version III—the final common pathway. Schizophr. Bull..

[3] Laruelle M (1996). Single photon emission computerized tomography imaging of amphetamine-induced dopamine release in drug-free schizophrenic subjects. Proc. Natl Acad. Sci. USA.

[4] Abi-Dargham A (1998). Increased striatal dopamine transmission in schizophrenia: confirmation in a second cohort. Psychiatry Res..

[5] Fusar-Poli P, Meyer-Lindenberg A (2013). Striatal presynaptic dopamine in schizophrenia, Part I: meta-analysis of dopamine active transporter (DAT) density. Schizophr. Bull..

[6] Howes OD (2012). The nature of dopamine dysfunction in schizophrenia and what this means for treatment. Arch. Gen. Psychiatry.

[7] Egerton A (2013). Presynaptic striatal dopamine dysfunction in people at ultra-high risk for psychosis: findings in a second cohort. Biol. Psychiatry.

[8] Howes OD (2011). Dopamine synthesis capacity before onset of psychosis: a prospective [18F]-DOPA PET imaging study. Am. J. Psychiatry.

[9] Halldin C (1995). Carbon-11-FLB 457: a radioligand for extrastriatal D2 dopamine receptors. J. Nucl. Med..

[10] Slifstein M (2015). Deficits in prefrontal cortical and extrastriatal dopamine release in schizophrenia: a positron emission tomographic functional magnetic resonance imaging study. JAMA Psychiatry.

[11] Piccini PP (2003). Dopamine transporter: basic aspects and neuroimaging. Mov. Disord..

[12] Jaber M, Jones S, Giros B, Caron MG (1997). The dopamine transporter: a crucial component regulating dopamine transmission. Mov. Disord..

[13] Giros B, Jaber M, Jones SR, Wightman RM, Caron MG (1996). Hyperlocomotion and indifference to cocaine and amphetamine in mice lacking the dopamine transporter. Nature.

[14] Chen KC (2013). Striatal dopamine transporter availability in drug-naive patients with schizophrenia: a case-control SPECT study with [(99m)Tc]-TRODAT-1 and a meta-analysis. Schizophr. Bull..

[15] Knable MB (1994). Quantitative autoradiography of dopamine-D1 receptors, D2 receptors, and dopamine uptake sites in postmortem striatal specimens from schizophrenic patients. Biol. Psychiatr..

[16] Pearce RK, Seeman P, Jellinger K, Tourtellotte WW (1990). Dopamine uptake sites and dopamine receptors in Parkinson’s disease and schizophrenia. Eur. Neurol..

[17] Czudek C, Reynolds GP (1989). [3H] GBR 12935 binding to the dopamine uptake site in post-mortem brain tissue in schizophrenia. J. Neural Transm..

[18] Dean B, Hussain T (2001). Studies on dopaminergic and GABAergic markers in striatum reveals a decrease in the dopamine transporter in schizophrenia. Schizophr. Res..

[19] Dean B, Bradbury R, Copolov DL (2003). Cannabis-sensitive dopaminergic markers in postmortem central nervous system: changes in schizophrenia. Biol. Psychiatr..

[20] Dean B (1999). Changes in serotonin2A and GABA(A) receptors in schizophrenia: studies on the human dorsolateral prefrontal cortex. J. Neurochem..

[21] Jung WH (2014). Unravelling the intrinsic functional organization of the human striatum: a parcellation and connectivity study based on resting-state FMRI. PLoS ONE.

[22] Martinez D (2003). Imaging human mesolimbic dopamine transmission with positron emission tomography. Part II: amphetamine-induced dopamine release in the functional subdivisions of the striatum. J. Cereb. Blood Flow. Metab..

[23] Scarr E, Udawela M, Dean B (2018). Changed frontal pole gene expression suggest altered interplay between neurotransmitter, developmental, and inflammatory pathways in schizophrenia. npj Schizophrenia.

[24] Semendeferi K, Armstrong E, Schleicher A, Zilles K, Van Hoesen GW (2001). Prefrontal cortex in humans and apes: a comparative study of area 10. Am. J. Phys. Anthropol..

[25] Dumontheil I (2014). Development of abstract thinking during childhood and adolescence: the role of rostrolateral prefrontal cortex. Dev. Cogn. Neurosci..

[26] Drevets WC, Furey ML (2010). Replication of scopolamine’s antidepressant efficacy in major depressive disorder: a randomized, placebo-controlled clinical trial. Biol. Psychiatr..

[27] Tully LM, Lincoln SH, Liyanage-Don N, Hooker CI (2014). Impaired cognitive control mediates the relationship between cortical thickness of the superior frontal gyrus and role functioning in schizophrenia. Schizophr. Res..

[28] Gracitelli CP (2015). Ophthalmology issues in schizophrenia. Curr. Psychiatry Rep..

[29] Matthews PR, Eastwood SL, Harrison PJ (2012). Reduced myelin basic protein and actin-related gene expression in visual cortex in schizophrenia. PLoS ONE.

[30] Meador-Woodruff JH (1997). Dopamine receptor transcript expression in striatum and prefrontal and occipital cortex. Focal abnormalities in orbitofrontal cortex in schizophrenia. Psychiatry Res..

[31] Schmitt U (1999). Chronic oral haloperidol and clozapine in rats: a behavioral evaluation. Neuropsychobiology.

[32] Tatsumi M, Groshan K, Blakely RD, Richelson E (1997). Pharmacological profile of antidepressants and related compounds at human monoamine transporters. Eur. J. Pharmacol..

[33] Arranz B, Marcusson J (1994). [3H]Paroxetine and [3H]citalopram as markers of the human brain 5-HT uptake site: a comparison study. J. Neural Transm./Gen. Sect..

[34] Javitch JA, Strittmatter SM, Snyder SH (1985). Differential visualization of dopamine and norepinephrine uptake sites in rat brain using [3H]mazindol autoradiography. J. Neurosci..

[35] Raffel DM, Chen W (2004). Binding of [3H]mazindol to cardiac norepinephrine transporters: kinetic and equilibrium studies. Naunyn Schmiedebergs Arch. Pharmacol..

[36] Barker EL (1998). High affinity recognition of serotonin transporter antagonists defined by species-scanning mutagenesis. An aromatic residue in transmembrane domain I dictates species-selective recognition of citalopram and mazindol. J. Biol. Chem..

[37] Zhou Y (2015). The selective impairment of resting-state functional connectivity of the lateral subregion of the frontal pole in schizophrenia. PLoS ONE.

[38] Arnsten AF, Girgis RR, Gray DL, Mailman RB (2017). Novel dopamine therapeutics for cognitive deficits in schizophrenia. Biol. Psychiatry.

[39] Dumontheil I, Burgess PW, Blakemore S-J (2008). Development of rostral prefrontal cortex and cognitive and behavioural disorders. Dev. Med. Child Neurol..

[40] Joyce JN, Lexow N, Bird E, Winokur A (1988). Organization of dopamine D1 and D2 receptors in human striatum: receptor autoradiographic studies in Huntington’s disease and schizophrenia. Synapse.

[41] Weinstein JJ (2017). Pathway-specific dopamine abnormalities in schizophrenia. Biol. Psychiatry.

[42] Simpson EH, Kellendonk C, Kandel E (2010). A possible role for the striatum in the pathogenesis of the cognitive symptoms of schizophrenia. Neuron.

[43] Rivest R, Falardeau P, Di Paolo T (1995). Brain dopamine transporter: gender differences and effect of chronic haloperidol. Brain Res..

[44] Reader TA, Ase AR, Huang N, Hébert C, van Gelder NM (1998). Neuroleptics and dopamine transporters. Neurochem. Res..

[45] Kapur S, VanderSpek SC, Brownlee BA, Nobrega JN (2003). Antipsychotic dosing in preclinical models is often unrepresentative of the clinical condition: a suggested solution based on in vivo occupancy. J. Pharmacol. Exp. Ther..

[46] Buchanan RW (2010). The2009 schizophrenia PORT psychopharmacological treatment recommendations and summary statements. Schizophr. Bull..

[47] Kapur S, Wadenberg ML, Remington G (2000). Are animal studies of antipsychotics appropriately dosed? Lessons from the bedside to the bench. Can. J. Psychiatry.

[48] Chang WH (2017). Unaltered dopamine transporter availability in drug-naive patients with schizophrenia after 6 months of antipsychotics treatment: a naturalistic study. J. Clin. Psychopharmacol..

[49] Mateos JJ (2007). Lower striatal dopamine transporter binding in neuroleptic-naive schizophrenic patients is not related to antipsychotic treatment but it suggests an illness trait. Psychopharmacology.

[50] Rothblat DS, Schneider JS (1997). Regionally specific effects of haloperidol and clozapine on dopamine reuptake in the striatum. Neurosci. Lett..

[51] Wright AM, Bempong J, Kirby ML, Barlow RL, Bloomquist JR (1998). Effects of haloperidol metabolites on neurotransmitter uptake and release: possible role in neurotoxicity and tardive dyskinesia. Brain Res..

[52] Fang J, Yu PH (1995). Effect of haloperidol and its metabolites on dopamine and noradrenaline uptake in rat brain slices. Psychopharmacology.

[53] Hsiao MC, Lin KJ, Liu CY, Tzen KY, Yen TC (2003). Dopamine transporter change in drug-naive schizophrenia: an imaging study with 99mTc-TRODAT-1. Schizophr. Res..

[54] Laakso A (2000). Striatal dopamine transporter binding in neuroleptic-naive patients with schizophrenia studied with positron emission tomography. Psychiatry Res..

[55] Schmitt GJ (2005). The striatal dopamine transporter in first-episode, drug-naive schizophrenic patients: evaluation by the new SPECT-ligand[99mTc]TRODAT-1. J. Psychopharmacol..

[56] Laakso A (2001). Decreased striatal dopamine transporter binding in vivo in chronic schizophrenia. Schizophr. Res..

[57] Sjoholm H, Bratlid T, Sundsfjord J (2004). 123I-beta-CIT SPECT demonstrates increased presynaptic dopamine transporter binding sites in basal ganglia in vivo in schizophrenia. Psychopharmacology.

[58] Kingsbury AE (1995). Tissue pH as an indicator of mRNA preservation in human post-mortem brain. Brain Res. Mol. Brain Res..

[59] Stan AD (2006). Human postmortem tissue: what quality markers matter?. Brain Res..

[60] Hill C (1996). Problem of diagnosis in postmortem brain studies of schizophrenia. Psychiatry Res..

[61] Roberts SB (1998). Confirmation of the diagnosis of schizophrenia after death using DSM-IV: a Victorian experience. Psychiatry Res..

[62] Foster P (1989). Neuroleptic equivalence. Pharm. J..

[63] Roth BL, Sheffler DJ, Kroeze WK (2004). Magic shotguns versus magic bullets: selectively non-selective drugs for mood disorders and schizophrenia. Nat. Rev. Drug Discov..

[64] Grant S, Fitton A (1994). Risperidone. Drugs.

[65] Paxinos G, Watson C (1986). The Rat Brain in Sterotaxic Coordinates, 2nd edn.

[66] Javitch JA, Blaustein RO, Snyder SH (1983). [3H]mazindol binding associated with neuronal dopamine uptake sites in corpus striatum membranes. Eur. J. Pharmacol..

[67] D’Agostino RB, Belanger A, D’Agostino RB (1990). A suggestion for using powerful and informative tests of normality. Psychiatry Res..

[68] Grubbs F (1969). Procedures for detecting outlying observations in samples. Technometrics.

[69] Cook RD, Weisberg S (1999). Applied Regression Including Computing and Graphics.

[70] Udovičić M, Baždarić K, Bilić-Zulle L, Petrovečki M (2007). What we need to know when calculating the coefficient of correlation?. Psychiatry Res..

