# Supplementary material for: Altered levels of dopamine transporter in the frontal pole and dorsal striatum in schizophrenia
Source: NPJ Schizophr. 2019 Dec 2;5:20. doi: 10.1038/s41537-019-0087-7 (PMC6888821; doi:10.1038/s41537-019-0087-7)
Supplement: Supplementary file 2 — Supplementary Infomation [file 41537_2019_87_MOESM2_ESM.pdf]

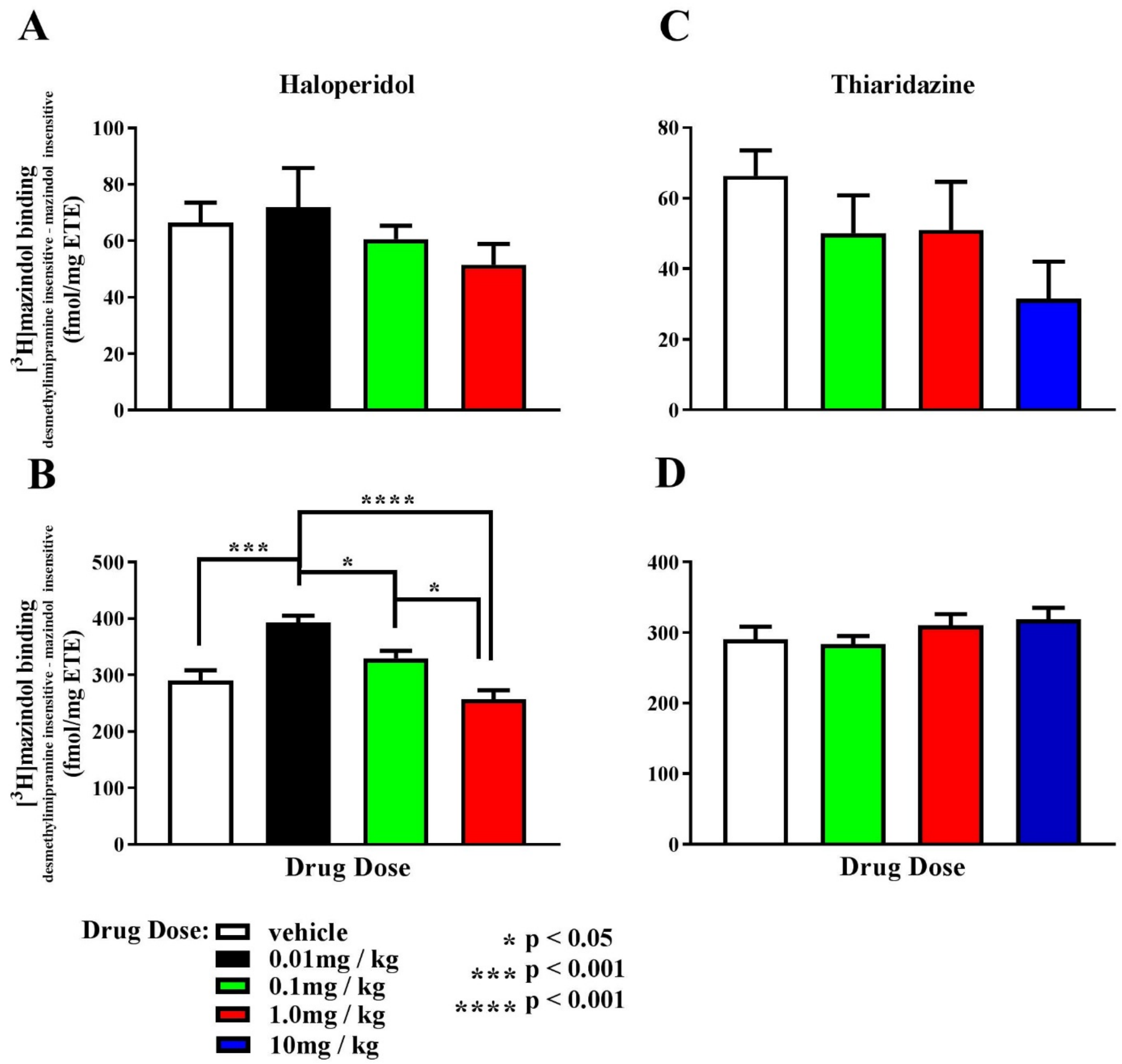

Supplementary Figure 1: The binding (mean ± SEM) of [^{3}H]mazindol to the cortex (A and C) and striatum (B and D) of rats treated for 3 months with varying doses of haloperidol (A and B) or thioridazine (C and D).

Supplementary Table 1: Demographic, pharmacological and CNS collection data for cases used in the study of dopamine transporters in the cortex and striatum from subjects with schizophrenia.

	Age (years)	Sex	pH	PMI (hr)	DOI (years)	Suicide	Cause of Death	Final recorded prescribed	FRADD	LEAP
**Controls**	43	M	6.45	30.5		N	Ischaemic heart disease			
	52	M	6.52	33.75		N	Cardiomegaly			
	48	F	5.89	24		N	Pulmonary embolus			
	76	F	6.01	53		N	Multiple organ failure, Septicaemia			
	71	M	6.11	59		N	Right ventricle rupture in operation			
	75	M	6.19	69.4		N	Acute myocardial infarction			
	55	M	6.69	30.5		N	Coronary Artery Atherosclerosis			
	66	M	6.47	71.75		N	Coronary Artery Atheroma			
	81	F	6.28	55		N	Ischaemic heart disease			
	63	F	6.55	50.25		N	Coronary Artery Atherosclerosis			
	69	F	6.32	38		N	Acute asthma			
	56	F	5.88	24		N	Cardiac tamponade			
	21	F	6.03	58		N	Myocarditis			
	64	M	6.59	69		N	Coronary Artery Atheroma			
	62	F	6.45	40		N	Ischaemic heart disease			
**Schizophrenia**	41	M	6.64	52	15	Y	Hanging	Fluphenazine decanoate	166	2490.0
	57	M	6.06	24	28	N	Coronary Artery Atheroma	Fluphenazine decanoate	150	4200.0
	38	F	6.43	20	17	Y	Burning	Fluphenazine decanoate	485	8245.0
	66	F	6.35	50	18	N	Rupture abdominal aortic aneurysm	Fluphenazine decanoate, Haloperidol	550	9900.0
	67	M	6.19	43.5	30	N	Lung cancer, Ischaemic heart disease			
	71	M	6.45	48	53	N	Aspiration of food	Thioridazine	150	7950.0
	61	M	6.01	45.5	18	Y	Hypovolemic shock	Risperidone	285	5130.0
	61	M	6.46	37.5	38	N	Ischaemic heart disease	Fluphenazine decanoate	745	28310.0
	72	F	6.48	58.5	37	N	Aspiration pneumonia	Chlorpromazine	25	925.0
	48	F	6.21	52.5	22	N	Pulmonary embolus	Fluphenazine decanoate, Chlorpromazine	700	15400.0
	76	F	6.28	52	22	N	Meningitis, Aspiration of food			
	59	F	6.19	44.5	35	N	Respiratory failure	Clozapine	632	22111.0

47	F	6.31	50	20	N	Pneumonia	Risperidone	600	12000.0
71	M	6.49	28.5	37	N	Ischaemic heart disease	Pimozide	400	14800.0
59	F	6.44	46	44	N	Congestive heart failure	Fluphenazine decanoate, Chlorpromazine	800	35200.0

Abbreviations: DOI = duration of illness, FRADD = final recorded antipsychotic drug dose expressed as chlorpromazine equivalents per day, N = no, PMI = postmortem interval, Y = yes.

Supplementary Table 2: Correlations between desmethylimipramine-insensitive mazindol-sensitive [^{3}H]mazindol binding in different regions of the human CNS.

Region		BA10	BA17	DPU	DCA	NAc
BA 10	r^2	*	0.05	0.18	0.06	0.01
	p		0.23	0.02	0.23	0.65
BA 17	r^2		*	0.04	0.02	0.13
	p			0.35	0.44	0.10
DPU	r^2			*	0.63	0.56
	p				<0.0001	<0.0001
DCA	r^2				*	0.50
	p					0.0002

Abbreviations: BA = Brodmann's area, DCA = dorsal caudate, DPU = dorsal putamen, NAc = nucleus accumbens.

Supplementary Table 3: Relationships between desmethylimipramine-insensitive mazindol-sensitive [^{3}H]mazindol binding and demographic, CNS collection and pharmacological data for cases used in this study.

CONTROLS

		BA10	BA17	DPU	DCA	NAc
Age	r^2	0.0006	0.001	0.001	0.02	0.04
	p	0.93	0.91	0.91	0.62	0.55
PMI	r^2	0.03	< 0.0001	0.02	0.01	0.03
	p	0.53	0.99	0.58	0.70	0.62
pH	r^2	0.05	0.007	0.15	0.20	**0.44**
	p	0.43	0.76	0.15	0.10	**0.01**

SCHIZOPHRENIA

		BA10	BA17	DPU	DCA	NAc
Age	r^2	0.004	0.01	0.07	0.06	0.05
	p	0.81	0.69	0.40	0.44	0.53
PMI	r^2	0.02	0.04	0.21	0.02	0.04
	p	0.64	0.48	0.14	0.69	0.56
pH	r^2	0.04	0.24	0.03	0.03	0.22
	p	0.45	0.08	0.58	0.62	0.15
DI	r^2	0.02	0.0001	0.14	0.27	0.008
	p	0.58	0.97	0.22	0.08	0.80
FRADD	r^2	**0.41**	0.03	0.0002	0.15	0.34
	p	**0.01**	0.59	0.96	0.22	0.06
LEAP	r^2	**0.47**	0.003	0.002	0.16	0.21
	p	**0.005**	0.85	0.88	0.20	0.15

Abbreviations: BA = Brodmann's area, DCA = dorsal caudate, DI = duration of illness, DPU = dorsal putamen, FRADD = final recorded dose of antipsychotic drug, LEAP = lifetime exposure to antipsychotic drug, NAc = nucleus accumbens, PMI = postmortem interval.
